# Post-abortion family planning utilization and associated factors in health facilities of Wolaita Zone, Southern Ethiopia: Mixed study

**DOI:** 10.1371/journal.pone.0267545

**Published:** 2022-06-03

**Authors:** Tizita Tekle Lencha, Addisu Alemayehu Gube, Molalegn Mesele Gessese, Mulugeta Tsegay Abadi

**Affiliations:** 1 School of Public Health, College of Medicine and Health Science, Wolaita Sodo University, Wolaita Sodo, Ethiopia; 2 School of Public Health, College of Medicine and Health Science, Dilla University, Dilla, Ethiopia; 3 Department of Midwifery, College of Medicine and Health Science, Wolaita Sodo University, Wolaita Sodo, Ethiopia; 4 Department of Maternity and Reproductive Health Nursing, College of Medicine and Health Science, Wolaita Sodo University, Wolaita Sodo, Ethiopia; University of Mississippi Medical Center, UNITED STATES

## Abstract

**Background:**

Unintended pregnancy due to disuse of family planning is the main cause of abortion globally. Women with a history of abortion are at higher risk of developing another unintended pregnancy, which may lead to repeated abortions and life-threatening complications. The immediate post-abortion period before women leave health institutions is a crucial time to provide family planning services. In Ethiopia, many women leave health facilities without using family planning methods. Therefore, this study aimed to determine the utilization of post-abortion family planning and its associated factors in health institutions in Wolaita Zone, Southern Ethiopia.

**Methods:**

Facility-based mixed cross sectional study was conducted between April 1 and June 30, 2018. A systematic sampling method was used to select the 408 participants. Seven key informants were selected for in-depth interviews and observations were made using a checklist. Data were collected through face-to-face interviews using a structured questionnaire. Data were entered into EPI INFO 3.5.1 and exported to SPSS 21 for cleaning and analysis. Bivariate analysis was employed and a P-value <0.25 was considered for the multivariable analysis. Qualitative data were coded and thematically analyzed to support the quantitative findings.

**Results:**

Data were obtained from 400 participants (response rate, 98%). The magnitude of post-abortion family planning was 67.3% [95% CI (62.8, 71.8)]. Marital status [AOR 95% CI 3.86(1.9, 7.8)], Good knowledge about post-abortion family planning [AOR 95% CI 2.48(1.22, 5.03)], Non-governmental health facility [AOR 95% CI 6.62(3.47, 12.6)] Counseling [AOR 95% CI 3.6(2.02, 6.4)] and husbands’ support [AOR 95% CI 3.21(1.81–5.7)] were significantly associated with Post-abortion family planning utilization.

**Conclusion:**

The utilization of post-abortion family planning was low in Wolaita Sodo health institutions. Marital status, knowledge of post-abortion family planning, use of services at non-governmental health facilities, counseling, and husbands’ support were determinants of post-abortion family planning.

## Introduction

Post-abortion family planning is an essential component of comprehensive abortion care that offers counseling and family planning methods immediately after, and within 48 hours of abortion, before fertility returns. It has been an element of high-quality abortion care since the early 1990s. The post-abortion time is an ideal time to provide family planning as the woman may be in better intention to use a method, she is known not to be pregnant, and she is in contact with a reproductive health care provider and may not return for a follow-up visit to receive family planning methods [1, 2].

Globally, approximately 40% of pregnancies are unintended, due to ineffective or non-use of family planning or method failure. Unintended pregnancy is the primary reason for abortion and half of these unintended pregnancies result in induced abortions [3].

In Sub-Saharan Africa, there are an estimated 5.5 million unsafe abortions every year. The unmet need for family planning is a major cause of abortions. More than 80% of the unintended pregnancies in developing countries occur in women with an unmet need for modern contraception. In sub-Saharan Africa and developing countries, the unmet need for family planning is one in four women in the reproductive age group of 15–49 years. Women will continue to face unintended pregnancies as long as their family planning needs are not met [4].

It is estimated that 90% of abortion-related morbidity could be prevented by the use of effective family planning methods, where effective family planning methods are available and widely used and the total abortion rate declines sharply [5]. Post-abortion family planning prevents unplanned pregnancies which can lead to repeated abortions. In 2012 the USAID technical advisory group declared that this was a high-impact practice in family planning. It is a component of comprehensive abortion care services [6].

Comprehensive abortion care services are a widely accepted public health strategy for minimizing maternal morbidity and mortality. Abortion care should be linked to comprehensive family planning to prevent unwanted pregnancies and repeated abortions from occurring [7]. WHO recommends that all women should receive contraceptive information and counseling regarding methods of post-abortion contraception, including emergency contraception, before leaving the health care facility [4].

Women who have had an abortion are at potential risk of having a subsequent abortion because they can get pregnant soon after the abortion; Studies that have examined return to ovulation post-abortion show that this can occur around 2–3 weeks following the abortion, but earlier in some women [8]. In addition, an interval of fewer than six months between an induced or incomplete abortion and a subsequent pregnancy is significantly associated with adverse pregnancy outcomes [9, 10].

In Ethiopia, as in other developing countries, low levels of family planning result in a high number of unintended pregnancies, which is the root cause of induced abortion. The first nationally representative study revealed an annual rate of 23 abortions per 1,000 women of reproductive age [11].

Following the revision of the abortion law which expanded the indications for safe abortion services, the Ministry of Health and its technical partners worked extensively to introduce a comprehensive abortion care model. It is an integrated set of sexual and reproductive health services that includes induced safe abortion for all legal indications, treatment of incomplete abortion and unsafe abortion, counseling, and provision of family planning and other reproductive health services as needed. Despite these efforts, service data have revealed a low rate of family planning utilization after abortion care [12].

## Materials and methods

### Study design and setting

A facility-based mixed-method study was conducted from April 1 to June 30, 2018, in Wolaita Sodo 380 km from Addis Ababa, the capital of Ethiopia. The town has a total population of 110,659 and 48% are women. It has one government referral hospital, one non-governmental hospital, one NGO RH center, three health centers, and 25 lower and 15 medium private clinics. Health service coverage of the town in 2010 was 89%.

### Population

The source population for the quantitative study was women who visited health facilities for abortion-related services, whereas the study population was comprised of women who visited health facilities for abortion-related services during the data collection period. All women in health facilities for abortion services were included in the study; however, those who were critically ill during the data collection period were excluded.

The study population for the qualitative study was the MCH head of the facility who was working at the studied facilities and the head of the town head office that were available during the data collection period.

### Sample size and sampling procedure

Sample size was determined using OpenEPI 3.03. A single population proportion was used with the following assumptions: 59.2% of the proportion of post-abortion family planning utilization [13], 95% confidence interval, power of 80%, 5% margin of error, 10% was added for non-response, and the final sample size was 408. The total sample size was allocated to six health facilities based on proportional allocation to size, which in turn was based on the average preceding year’s three-month client flow for abortion care services at each health facility. The study participants were selected using a systematic random sampling method by calculating the sampling interval, which was two (k = 2). The first client was selected as the study participant by using a simple random (lottery) method.

Six MCH heads (abortion unit heads of hospitals) and one maternal health department head of the Sodo town health office were selected based on their knowledge and sufficient information about post-abortion family planning services. Two observations at each facility were carried out to observe client-provider interactions and counseling.

### Study variables

#### Dependent variable

Is the utilization of post-abortion family planning. It is the provision of any family planning method immediately after an abortion-related care procedure for women visiting a health facility.

#### Independent variables

The independent variables were categorized as socio-demographic characteristics (age, marital status, educational level, occupation, and residence), facility and provider-related factors (service affordability, method choice, counseling), reproductive health-related factors: gravidity, fertility plans, history of FP use, previous abortion history, reason for current abortion), knowledge and attitude related factors (Attitude towards post-abortion family planning services, knowledge related to the risk of abortion, knowledge of contraceptive methods) and decision-making related factors (decision on own self, husband/partner’s support).

Fifteen family planning knowledge-related questions were used to measure the knowledge of post-abortion family planning. Respondents who answered more than the mean value were considered to have “Good knowledge" whereas respondents who answered below the mean were considered to have “Poor knowledge”. Ten questions were used to measure attitudes toward post-abortion family planning service provision. Respondents who answered less than the mean value were labeled to have “Negative attitude” towards post-abortion family planning services and those who scored above the mean were categorized to have “Positive attitude” (14).

### Data collection tools and procedure

Quantitative data were collected through an interviewer-administered client exit interview using a pretested structured questionnaire, adapted from a previous study to assess socio-demographic, reproductive health, Knowledge, and attitude toward the service and decision-making power-related factors [14]. The questionnaire prepared in English was translated into Amharic and re-translated back into English. The reliability of the questionnaire was verified and Cronbach’s alpha value was 0.836 for the knowledge questions and 0.809 for the attitude questions. Data were collected by six trained nurses and two health officers were assigned to supervision.

An in-depth interview guide was used to interview key informants from health facilities for qualitative data [14]. Structured observations were conducted to observe client-provider interactions. A data collector trained on the PAFP collected observational data using a structured service observation checklist.

### Data quality control

The questionnaire was pre-tested before implementation in 5% of the total sample estimated for this study in Areka health center and appropriate revisions were made to the tool based on the pre-test results. Two days of intensive training was provided to data collectors and supervisors on the objective of the study, data collection process, and relevance of the study to both data collectors and supervisors before actual data collection. In addition to supervisors, the principal investigator was actively involved in the supervision of data collection and the completed questionnaire was cross-checked daily for completeness. Data collectors were supervised at each site and regular communication was held between the data collectors, supervisors, and the principal investigator. The data were checked on-site for completeness. In-depth interviews were conducted in places where participants were more comfortable. Before coding, the data were familiarized to avoid missing ideas.

### Data processing and analysis

The collected data was cleaned, coded, and entered into Epi info 3.5.1and exported to SPSS V 21.0 statistical software package for analysis. Descriptive analyses were performed to determine the distribution of variables. Variables with a p-value <0.25 during bivariate analysis were used for multivariable analysis. Adjusted Odds Ratios (AORs) and 95% Confidence Intervals (CIs) were calculated for each independent variable. In all cases P-value <0.05 was considered, statistically significant. Multi-collinearity was checked using the tolerance and variance inflation factor (VIF) which was less than five. Model fitness was checked using the Hosmer-Lemeshow goodness of fit test. Qualitative data were transcribed and translated. It was then coded and analyzed thematically to supplement the quantitative findings.

### Ethical consideration

Ethical clearance was obtained from the Arba Minch University College of Medicine and Health sciences ethical review board and a formal letter was obtained from the population and family health department and delivered to the town health office before the study commencement. Health facilities were approached using a formal letter written by the town’s health office.

All study participants were well informed about the benefits of the study along with their right to refuse before proceeding with the questions. Participants were informed about the nature of the study, its possible benefits, and any possible harm. Written consent and assent was obtained from all participants.

The privacy and confidentiality of the study participants were assured by taking them into separate rooms for interviews and the information provided by each respondent was kept confidential.

## Results

### Socio-demographic characteristics

Four hundred women were included in this study yielding, a response rate of 98%. The majority of the clients, 184(46%) were in the age group of 18–24 years and the mean age was 24.4 years with a standard deviation of ± 3.9 years.

Of the respondents, 205(51.3%) had an educational level of tertiary education or higher, 247(61.8%) were single and 123(30.8%) were students. The monthly income for most of the clients 176(44%) was reported to be less than 500 Ethiopian birr and 312(79.5%) were urban residents [Table 1].

**Table 1:** Socio-demographic and economic characteristics of post-abortion clients, Wolaita Sodo, 2019.

Variables	Category	Number of clients (%)
Age	< 18 years	35(8.8%)
	18–24 years	184(46%)
	25–30 years	155(38.8%)
	> 30 years	26(6.5%)
Marital Status	Single	247(61.8%)
	Married	140(35%)
	Separated	13(3.3%)
Educational level	Illiterate	8(2%)
	Primary (1–8)	58(14.5%)
	Secondary (9–12)	129(32.3%)
	Tertiary and above	205(51.3)
Educational Level of Husband/Partner	Illiterate	3(0.8%)
	Primary (1–8)	27(6.8)
	Secondary (9–12)	165(41.3)
	Tertiary and above	205(51.3)
Occupation	Housewife	65(16.3%)
	Gov. employed	88(22%)
	NGO or private Org employed	60(15%)
	Student	123(30.8%)
	Daily laborer	30(7.5%)
	Merchant	34(8.5%)
Occupation of the husband/partner	Farmer	65(16.3%)
	Gov. employed	124(31%)
	NGO or private Org employed	31(7.8)
	Student	87(21.8)
	Merchant	60(15%)
	Daily Laborer	33(8.3%)
Average monthly income(ETB)	<500	176(44%)
	500–1000	72(18%)
	>1000	152(38%)
Place of residence	Rural	82(20.5%)
	Urban	318(79.5)

### Reproductive history of the clients

Of the respondents, 219(54.8%) have a history of previous pregnancy and from those who had a history of previous pregnancy, 124(31%) have a history of previous abortion. Fifty-four percent of the clients had more than two abortions including the current one. Most of the clients were nulliparous 181(45.3%). Of the respondents, 250(62.5%) utilized the SAC service and the remaining 150(37.5%) used PAC services. Of the service visitors, 53(35.34%) had abortions because of unwanted pregnancies.

Of the respondents, 392(98%) were interested in having an additional child in the future but most of them didn’t plan when to have a child 229(57.3%) and some of them 34(8.5%) wanted a child as soon as possible [Table 2].

**Table 2:** Reproductive history of post-abortion clients, Wolaita Sodo, 2019.

Variables	Category	Number of clients (%)
**History of previous pregnancy**	Yes	219(54.8%)
	No	181(45.3%)
**History of previous abortion**	Yes	124(31%)
	No	276(69%)
**Frequency of previous abortion**	Two times	56(45.16)
	More than two times	68(54.8%)
**Type of abortion service**	SAC	250(62.5%)
	PAC	150(37.5%)
**Reason for SAC service**	Rape	3(1.2%)
	Medical Condition	32(12.8%)
	Unwanted Pregnancy	215(86%)
**Reason for PAC service**	Taking the drug without prescription	28(18.67%)
	Started spontaneously	97(64.7%)
	Taking traditional medicine	25(16.67%)
**Interest to have an additional child**	Yes	392(98%)
	No	8(2%)
**Time plan to get pregnant again**	Want to get pregnant soon	34(8.7%)
	Within one year	46(11.7%)
	After one year	83(21.2%)
	Not planned yet	229(58.4%)

### Health service and contraceptive history

Most study participants 231 (57%) utilized PAFP in NGO health facilities. Of the total respondents, 103(25.8%) had a previous history of family planning counselling 398(99.25%), and the majority of respondents 341(85.3%) had a previous history of family planning use. Of the respondents, 335(83.8%) had a good knowledge of post-abortion family planning. Most respondents 218(54.5%) had husband/partner support for family planning.

Of the respondents, 279(69.8%) counselled on post-abortion family planning after providing abortion care services [Table 3].

**Table 3:** Health service and contraceptive history of post-abortion clients, Wolaita Sodo, 2019.

Variables	Categories	Number
**Type of health facility**	Government	108(27%)
	Non-Government	231(57%)
	Private	61(15.3%)
**History of FP counseling before**	Yes	103(25.8%)
	No	297(74.3%)
**History of contraceptive use before the current pregnancy**	Yes	341(85.3%)
	No	59(14.8%)
**Decision-maker to use FP**	Me And my husband/partner	166(41.5%)
	Only my husband	44(11%)
	Only me	190 (47.5%)
**Husband/partner support**	Yes	218(54.5%)
	No	182(45.5%)
**Knowledge related to PAFP**	Yes	335(83.8%)
	No	65(16.3%)
**Attitude to PAFP service**	Positive	278(69.5%)
	Negative	122(30.5%)
**Counseled about PAFP today**	Yes	279(69.8%)
	No	121(30.2)
**Using FP after the abortion service today**	Yes	269(67.3%)
	No	131(32.8%)
**Reason for non-users**	Not planned to have sex	61(46.5%)
	Not getting information about FP	15(11.4%)
	Husband/partner not around	45(34.3%)
	Want to be pregnant soon	18(13.7%)
	Fear of side effects	14(10.7%)
	Opposition from partner	16(12.2%)
	Method of choice is not available	12(9.16%)
**Type of family planning method provided**	Pill	25(9.3%)
	Injectable	134(49.8%)
	Implants	82(30.5%)
	IUCD	23(8.5%)
	Condom	5(1.86%)
**Taking Method of choice**	Yes	240 (89.2%)
	No	29(10.8%)
**Reason for a not-using method of choice**	Didn’t find the method of choice	13(44.83%)
	No trained provider	6(20.7%)
	Provider Refusal	10(34.5%)

The major reasons for not using FP were no plan to have sex 61(46.5%) and 45(34.3%) wanted to be pregnant soon 18(13.43%).

**Fig 1:**
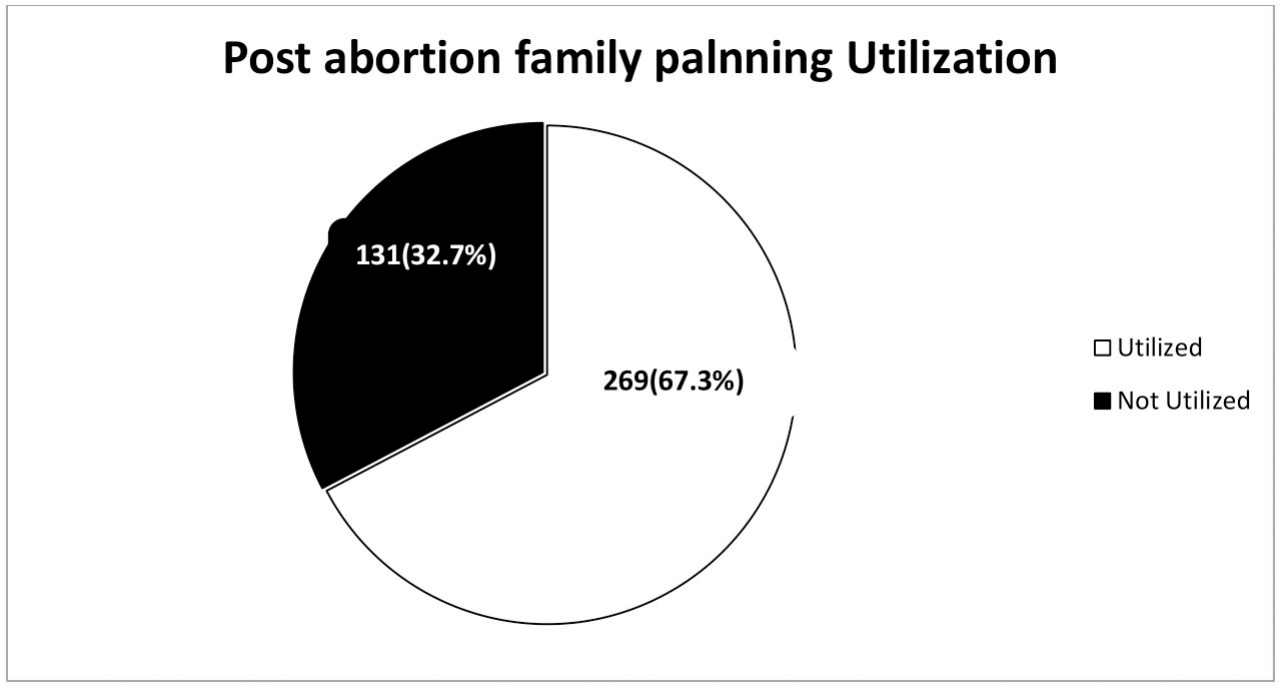
Level of post-abortion family planning utilization in health facilities among abortion clients in Wolaita Sodo town, 2019.

Among respondents, 67.3% used the post-abortion family planning method [Fig 1]. The majority of respondents, 240 (89.2%) used the method of their choice. The major reasons for not using the method of choice were 13 (44.83%) absence of the method of choice and 10 (34.5%) providers’ refusal to provide the method choice of the mother.

**Fig 2:**
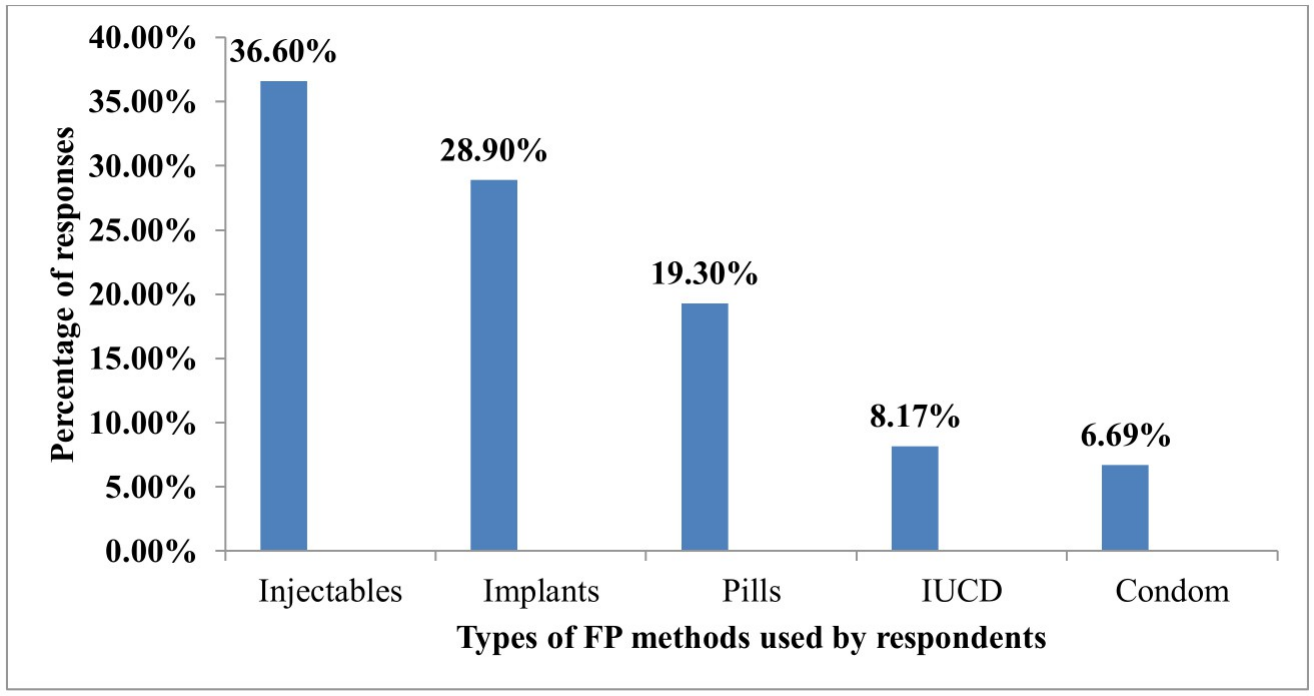
Types of modern PAFP methods utilized from health facilities in Wolaita Sodo, 2019.

Among the participants who underwent post-abortion family planning, 134(49.8%) used the injectable method followed by 82(30.5%) implants [Fig 2].

### Factors associated with utilization of post abortion family planning

In bivariate analysis, a significant association was observed between post-abortion family planning utilization and Marital status, Monthly income, Type of health facility, history of previous pregnancy, history of previous abortion, history of using modern FP methods, knowledge of PAFP, husbands’ support, and counseling [Table 4].

**Table 4:** Factors associated with PAFP utilization among post-abortion clients in health facilities in Wolaita Sodo town, 2019.

Variables	Categories	PAFP utilization		COR(95% CI)	AOR(95% CI)
		Yes	No		
**Marital status**	Single	139	108	Ref	Ref
	Married	125	15	6.47(3.58, 11.7)	3.86(1.9, 7.8) ***
	Separated	5	8	0.48(0.15, 1.53)	1
**Monthly income**	<500	111	65	Ref	Ref
	500–1000	56	16	2.05(1.09, 3.86)	1
	>1000	102	50	1.19(0.75, 1.89)	1
**Previous pregnancy history**	Yes	162	57	1.96(1.29, 3)	1
	No	107	74	Ref	Ref
**Previous abortion history**	Yes	99	25	2.47(1.49, 4.07)	1
	No	170	106	Ref	Ref
**History of FP using**	Yes	238	103	2.09(1.19, 3.66)	1
	No	31	28	Ref	Ref
**Knowledge**	Yes	241	94	3.38(1.96, 5.85)	2.48(1.22, 5.03) *
	No	28	37	Ref	Ref
**Counseling**	Yes	216	63	4.39(2.79, 6.94)	3.6(2.02, 6.4)***
	No	53	68	Ref	Ref
**Husband support**	Yes	168	50	2.7(1.75, 4.14)	3.21(1.81–5.7)***
	No	101	81	Ref	Ref
**Type of health facility**	Government	48	60	Ref	Ref
	Non-Government	188	43	5.46(3.3, 9.05)	6.62(3.47,12.6)***
	Private	33	28	1.47(0.78, 2.77)	1

* = p-value<0.05,

*** = p-value 0.000

Multivariable logistic regression analysis revealed that marital status, knowledge of PAFP, husband/partner support, counseling, and type of health facility were significantly associated with PAFP utilization [Table 4].

Married women were 3.8 times more likely to utilize family planning than single women [AOR 3.86(1.9, 7.8)]. Similarly, clients who have husband/partner support were 3.21 times more likely to utilize family planning methods than those who had no husband/partner support to use family planning methods [AOR 3.03(1.72–5.33)].

Women who received abortion services at NGO health facilities were 6.62 times more likely to adopt family planning methods when compared to government health facilities [AOR 6.62(3.47, 12.6)]. This finding is supported by the qualitative findings.

A 38 years old female service provider said” *…there are an unbalanced number of health professionals as compared to patients/clients*, *especially in government health facilities*. *This minimizes the contact time between the client and service provider and leads to poor client-provider interaction during counseling which is one of the main problems that lowers service Utilization*.”

The odd of using post-abortion family planning was 2.48 times higher in clients who have good knowledge about post-abortion family planning than clients who have poor knowledge [AOR 2.48(1.22, 5.03)].

Clients who had received post-abortion family planning counseling were 3.6 times more likely to utilize post-abortion family planning compared to clients who didn’t receive counseling [AOR 3.6(2.02, 6.4)]. Qualitative findings also support this finding.

A 40 years old male service provider said”… *negligence on health professionals while providing the service is seen on some of our health professionals while counseling and approaching the clients which can make clients leave the facility without the FP method*.”

The scarcity of trained service providers in post-abortion family planning has been mentioned as a problem in almost all institutions, especially in governmental health facilities.

A 32 years old male service provider said that “…. *health professionals who were specifically trained in abortion-related services were insufficient to address the number of clients*. *Service providers that don’t undergo training may not counsel clients accordingly*. *Good counseling requires knowledge of how to approach clients and training improves this ability*.”

Poor client-provider interactions were observed during most counseling sessions. The majority of clients counseled with poor client-provider interactions during the observation left the facility without family planning methods.

## Discussion

In this study, the rate of post-abortion family planning utilization was 67.3%. Marital status, counseling on PAFP, type of health facility, husband/partner support, and knowledge of PAFP were associated with PAFP utilization.

The magnitude of PAFP utilization revealed in this study is consistent with the findings of studies conducted in Jimma (71.5%) [14] and Central Tigray (70.9%) [15] and higher than those reported in Debre Markos (59.2%) [13], Dessie (47.5%) [16] Gurage (56.5%) [17] and Tigray Shire (61.5%) [18]. Regarding studies conducted in Debre Markos and Dessie, a possible reason might be the time gap in which health services have improved over time, however in Guraghe and Tigray studies the respondents were only from public health facilities. However, this rate was lower than that reported by Addis Ababa (90.6%) [19]. The reason for this might be that the respondents in Addis Ababa had a better knowledge of PAFP due to they had more media access than those living in semi urban areas.

This result was also lower than that reported in studies in Brazil (97.4%) [20] and Nepal (83%) 21]. The disparity might be, more than ninety percent of respondents in Nepal and Brazil have good knowledge of post-abortion family planning. Another possible reason may be poor client-provider interactions during counseling. As mentioned in the qualitative section of this study poor client-provider interaction may be one of the causes of low utilization.

Married women were 3.8 times more likely to utilize post-abortion family planning than single women. A study conducted in Jimma also revealed that married women were 6.7 times more likely to utilize post-abortion family planning than unmarried women [14]. However, a study conducted in Debre Markos showed that married women were 44% less likely to use family planning than single women [13]. One possible reason might be that married women are influenced by their husbands.

In this study, counseling was significantly associated with utilization of post-abortion family planning. Clients who received counseling were 3.6 times more likely to utilize PAFP than their counterparts. A study conducted in Debre Markos and Tigray (Shire) was also in line with this study: women who received family planning counseling were 3.5 times and 4 times more likely to use PAFP respectively [13, 18]. This reveals that the immediate post-abortion period is crucial for providing family planning advice because women are more ready to receive messages. This finding was also supported by a qualitative study in which all participants agreed that counseling with good interaction had a pertinent influence on a client’s utilization interest.

Non-governmental health facilities were significantly associated with post-abortion family planning. Clients from NGO clinics were 6.62 times more likely to utilize PAFP when compared to government health facilities where the utilization was less than half (46.7%). Similarly, a study from Central Tigray showed that individuals served in NGOs were 6.7 times more likely to receive contraceptives than those served in public facilities [15]. This might be because service providers in NGO clinics offer better counselling on post-abortion family planning. In contrast, studies conducted in Tigray Shire show that women who received abortion services at public health institutions were 5.9 times more likely to utilize family planning than their counterparts [18]. This disparity might be because the study in Tigray Shire includes only public and private clinics and in this study, all NGOs were also included. A possible reason might be that there is a work overload due to a large number of patient flows in public health facilities: which decreases the counseling time of the client and service provider. A qualitative study supports the finding that Public health facilities have high workloads.

This study found a significant association between husband/partner support and post-abortion family planning. Clients whose husbands/partners supported the use of post-abortion family planning were 3.21 times more likely to utilize PAFP than their counterparts were. This study is in line with a study in Tigray where women who had male opposition were 77.7% less likely to utilize FP before leaving the facility than their counterparts [15]. A study in Egypt also revealed that husbands’ disapproval was the reason for not receiving post-abortion family planning [22]. This finding is consistent with qualitative findings.

Clients with good Knowledge of post-abortion family planning were 2.48 times higher odds of using it than those with poor knowledge. A study conducted in Shire, Addis Ababa, Egypt, Brazil and Nepal showed that knowledge about PAFP was significantly associated with post-abortion family planning. One possible reason might be that clients with good knowledge increase their awareness and intention to use post-abortion family planning [18–22]. As explained in the qualitative part of this study most of the participants also agreed with the quantitative findings providing information and education regarding post-abortion family planning gives the knowledge to increase the utilization of post-abortion family planning services.

## Strength and limitations of the study

The study included all facilities that provide abortion services in the town (NGOs, government, and private facilities). A qualitative design was used to supplement the quantitative findings for some of the variables.

Social desirability bias might be a limitation of this study because of its sensitivity. To curb this problem, clients were informed that all their words would be kept confidential and were told the importance of their cooperation and genuine comments to improve PAFP services.

## Conclusion

The utilization of post-abortion family planning in health institutions of Sodo town is unsatisfactory. Approximately one-third of the post-abortion clients left health facilities without modern family planning methods. Being married, receiving family planning counseling, the type of health facility, husband/partner support, and knowledge about post-abortion family planning were found to be significant factors for post-abortion family planning utilization. Scarcity of trained service providers, work overload, service provider negligence, knowledge of PAFP of clients, and husband/partner refusal were mentioned as barriers to post-abortion family planning utilization. Poor client-provider interactions were also observed during counseling.

## Supporting information

S1 File English version questionnaire new. (DOCX)

S2 File Amharic version questionnaire new. (DOCX)

## Abbreviations

AOR: Adjusted Odds Ratio
CAC: Comprehensive Abortion Care
CI: Confidence Interval
COR: Crude Odds Ratio
FP: Family Planning
HF: Health Facility
IEC: Information Education Communication
IUCD: Intrauterine Contraceptive Device
NGO: Non-Governmental Organization
PAC: Post Abortion Care
PAFP: Post Abortion Family Planning
RH: Reproductive Health
SAC: Safe Abortion Care
SNNPR: Southern Nations, Nationality and Peoples Region
WHO: World Health Organization

## References

[1] World Health Organization. Medical eligibility criteria for contraceptive use. In: Health DoR, editor. Fourth edition ed. Geneva, Switzerland: WHO Press; 2010.

[2] Huber D, Curtis C, Irani L, Pappa S, Arrington L. Postabortion Care: 20 Years of Strong Evidence on Emergency Treatment, Family Planning, and Other Programming Components. Glob Health Sci Pract. 2016 Sep 29;4(3):481–94. doi: 10.9745/GHSP-D-16-00052 27571343 PMC5042702

[3] Sedgh G, Singh S, Hussain R. Intended and unintended pregnancies worldwide in 2012 and recent trends. Stud Fam Plann. 2014 Sep;45(3):301–14. doi: 10.1111/j.1728-4465.2014.00393.x . 25207494 PMC4727534

[4] World Health Organization. Safe abortion: Technical and policy guidance for the health system. 2nd edition ed. Geneva: WorldHealthOrganization; 2012.

[5] S DJ, Ashford LS, Vlassoff M. Adding it up: the costs and benefits of investing in family planning and maternal and newborn health. New York:: Guttmacher Institute and UNFPA; 2009.

[6] USAID. High Impact Practices in Family Planning (HIP). Postabortion family planning: strengthening the family planning component of postabortion care. Washington DC: USAID; 2012.

[7] Vélez LF, Sanitato M, Barry D, Alilio M, Apfel F, Coe G, et al. The role of health systems and policy in producing behavior and social change to enhance child survival and development in low- and middle-income countries:J Health Commun. 2014;19 Suppl 1(sup1):89–121. doi: 10.1080/10810730.2014.939313 . 25207449 PMC4205911

[8] Stoddard A, Eisenberg DL. Controversies in family planning: timing of ovulation after abortion and the conundrum of postabortion intrauterine device insertion. Contraception.2011;84(2):119–21. Epub 2011 Feb 11. . 21757051 10.1016/j.contraception.2010.12.010 PMC3443686

[9] Makhlouf MA, Clifton RG, Roberts JM, Myatt L, Hauth JC, Leveno KJ, et al. Eunice Kennedy Shriver National Institute of Child Health Human Development Maternal-Fetal Medicine Units Network. Adverse pregnancy outcomes among women with prior spontaneous or induced abortions. Am J Perinatol. 2014;31(9):765–72. 24347257 10.1055/s-0033-1358771 PMC4061262

[10] Bigelow CA, Bryant AS. Short Interpregnancy Intervals: An Evidence-Based Guide for Clinicians. Obstet Gynecol Survey. 2015;70(7):458–64. doi: 10.1097/OGX.0000000000000195 . 26185917

[11] Singh S, Fetters T, Gebreselassie H, Abdella A, Gebrehiwot Y, Kumbi S, et al. The estimated incidence of induced abortion in Ethiopia, 2008. Int Perspect Sex Reprod Health. 2010 Mar;36(1):16–25. . 20403802 10.1363/ipsrh.36.016.10

[12] Samuel M, Fetters T, Desta D. Strengthening Postabortion Family Planning Services in Ethiopia: Expanding Contraceptive Choice and Improving Access to Long-Acting Reversible Contraception. Glob Health Sci Pract. 2016 Aug 18;4 Suppl 2(Suppl 2):S60–72. doi: 10.9745/GHSP-D-15-00301 . 27540126 PMC4990163

[13] Lealem K A E, Kassa H. Utilization of Post Abortion Contraceptive and Associated Factors among Women who Came for Abortion Service in Debre Markos. Journal of family medicine and disease prevention. 2015;1(4):2469–5793.

[14] Erko EK, Abera M, Admassu B. Safe Abortion Care, Utilization of Post Abortion Contraception and associated Factors, Jimma Ethiopia. J Women’s Health Care 2016 5(4): 321.

[15] Hagos G., Tura G., Kahsay G. et al. Family planning utilization and factors associated among women receiving abortion services in health facilities of central zone towns of Tigray, Northern Ethiopia: BMC Women’s Health 18, 83 (2018). doi: 10.1186/s12905-018-0582-4 29871631 PMC5989472

[16] Abebe Ayele Mamo, Wudu Kassaw Mesfin, Estifanos Shewangashaw Nathan, “Postabortion Contraception Acceptance and Associated Factors in Dessie Health Center and Marie Stopes International Clinics, South Wollo Northeast, Amhara Region, 2017”, International Journal of Reproductive Medicine, 2019, 1327351, 10, 2019. doi: 10.1155/2019/1327351 31531342 PMC6719265

[17] Tesfaye Gezahegn & Oljira Lemessa. Post Abortion Care Quality Status in Health Facilities of Guraghe Zone, Ethiopia. Reproductive health. 2013.10. 35. doi: 10.1186/1742-4755-10-35 23875945 PMC3726452

[18] Moges Y, Hailu T, Dimtsu B, Yohannes Z, Kelkay B. Factors associated with uptake of post-abortion family planning in Shire town, Tigray, Ethiopia. BMC Res Notes. 2018;11(1):928. doi: 10.1186/s13104-018-4029-7 30591074 PMC6307259

[19] Asrat M, Bekele D, Sarah D, et al. Post-abortion contraceptive acceptance and choice determinants among women receiving abortion care at Saint Paul’s hospital, Addis Ababa, Ethiopia. Ethio J Reprod Health (EJRH). 2018;10(1):35–48.

[20] Ferreira A.L.C., Souza A.I., Lima R.A. et al. Choices on contraceptive methods in post-abortion family planning clinic in the northeast Brazil. Reprod Health. 2010. 7, 5. doi: 10.1186/1742-4755-7-5 20459754 PMC2883537

[21] Khanal V, Joshi C, Neupane D, Karkee R. Practices and perceptions on contraception acceptance among clients availing safe abortion services in Nepal. Kathmandu Univ Med J (KUMJ). 2011. 9(35):179–84. doi: 10.3126/kumj.v9i3.6301 . 22609503

[22] Mahmoud Ghadah A. and Byomy Soad S. Fertility awareness and family planning use among post abortion women in Egypt. Life Sci J 2013;10(1):143–150 (ISSN:1097-8135)

