# Supplementary material for: Post-abortion family planning utilization and associated factors in health facilities of Wolaita Zone, Southern Ethiopia: Mixed study
Source: PLoS One. 2022 Jun 3;17(6):e0267545. doi: 10.1371/journal.pone.0267545 (PMC9165889; doi:10.1371/journal.pone.0267545)
Supplement: S1 File — (DOCX) [file pone.0267545.s001.docx]

## Consent form

## Title: Post Abortion Family Planning Utilization and Associated Factors in health facilities of Wolaita Zone, Southern Ethiopia: Mixed Study.

**Name of Principal Investigator**: Tizita Tekle (BSc)

**Name of the organization**: Arba Minch University

**Introduction:**

Greeting:

Hello! My name is ---------------------I am working in research team of Arba Minch University, Thank you for agreeing to talk to me today This is a study to be conducted with objective of identifying factors which may encourage or discourage women of reproductive age to utilize post abortion family planning in health facilities. As the study is directly related to women of reproductive age, you are one of the women who are selected to participate in this study, therefore you are kindly requested to participate in this study and provide the information required from you.

**Confidentiality:** “I’m going to ask you some personal questions related to contraceptive use and abortion. There is not necessarily any right or wrong answer. I would like to ask you share your views as freely and completely as possible. We will protect the confidentiality of your responses to the best of our ability. We need your signature but your name will not be written on this form and will never be used in connection with any of the information you tell me.

**Incentives:** You will not be provided any payment to take part in this study.

**Risks and/or Discomfort:** There is no risk in participating in this research project.

**Right to refuse or withdraw**: Your participation in this study is completely on voluntary bases and you have the right to refuse from participation. Your decision on whether or not to participate in the interview will not affect the health care you receive at this facility. You do not have to answer any questions that you do not want to answer, and you may end this interview at any time you want to.

**Benefits of the study:** your honest answers to these questions will help us improve our understanding of the problem/gap on the services. We would greatly appreciate your participation in this interview. It will take about 10-15 minutes

**Person to contact**: If you have any questions or concerns or more explanation about the research, you can contact at the following address.

Name: Tizita Tekle

Telephone number: 0934237207 Email:tizita23t@gmail.com

If you are willing to participate in this study, we are pleased you to sign. Are you willing to participate?

Yes ---------------

No -----------------

If yes, Signature__________________

Then continue interviewing

If No, thank and stop interviewing

Name of interviewer--------------- sign ----- Date of interview---------

Name of the supervisor --------------------- Sign --------------- Date-------

# Annex 2: Questionnaire

| **QUESTIONER FOR POST-ABORTION CLIENTS TO ASSESS CONTRIBUTING FACTORS FOR POST-ABORTION CONTRACEPTION.** | | | | | | | | | | | | | | | |
| --- | --- | --- | --- | --- | --- | --- | --- | --- | --- | --- | --- | --- | --- | --- | --- |
| Serial no. | | | Question | | | | Respond | | | | | | Remark | | |
| 1.Socio demography related factor | | | | | | | | | | | | | | | |
| 1.1 | | What is your age? | | | |  | | | | | | | |  | |
| 1.2 | | Where is your place of residence? | | | |  | | | | | | | |  | |
| 1.3 | | What is your marital status? | | | | 1. Single 2. Regular partner but not married 3. Married 4. Divorced 5. Widowed | | | | | | | |  | |
| 1.4 | | What is the level  income in month | | | |  | | | | | | | |  | |
| 1.5 | | What is your educational level? | | | | 1. Illiterate  2.Primary  3.Secondary  4.Tertiary(12 and above) | | | | | | | |  | |
| 1.7 | | What is the educational level of your husband/partner? | | | | 1. Illiterate  2.Primary  3.Secondary  4.Tertiary(12 and above) | | | | | | | |  | |
| 1.8 | | What is your  Occupation? | | | | 1.Housewife  2. Government employee  3. NG employee  4. Student  5. Daily laborer  6. Merchant  7. Others (specify) | | | | | | | |  | |
| 1.9 | What is your husbands/partners  Occupation? | | | | | 1. Farmer  2. Government employee  3. NG employee  4. Student  5. Daily laborer  6. Merchant  7. Others (specify) | | | | | | | |  | |
| 2.Family planning and Health service related questions | | | | | | | | | | | | | | | |
| 2.1 | | | | Have you ever counseled about post abortion  Family planning? | | | | 1.yes  2.no | | | | | | |  |
| 2.2 | | | | Have you ever used any method of family planning? | | | | 1. Yes  2. No | | | | | | |  |
| 2.7 | | | | Did the service provider discuss with you on FP options today? | | | | 1. Yes  2. No | | | | | | |  |
| 2.8 | | | | Have you received any FP methods from this facility today? | | | | 1.yes  2.no | | | | | | | If no 2.10 |
| 2.9 | | | | If Yes to Q 2.7, Which FP method did you receive from this facility? | | | | 1. Oral contraceptives  3. Injectable 4. Implant  5. Condoms 6. IUCD  7.Permanentmethods 8.Others_________ | | | | | | |  |
| 2.10 | | | | If no to 2.8, what is the reason? | | | | 1. I didn’t get information on FP service  2. I want to get pregnant soon  3. Method of my choice is not available  4. My husband/Partner is away from me.  5. I have no plan to have sexual contact again  6.fear of drug side effect  7.other specify | | | | | | |  |
| 2.11 | | | | Have you got the method you chose? | | | | 1. Yes  2. No | | | | | | |  |
| 2.12 | | | | If No to Q 2.11 What was the reason for not obtaining your method of choice? | | | | 1.Method is not available  2.There is no trained provider  3. Provider refusal  4.Other | | | | | | |  |
| 3.Reproductive health related data | | | | | | | | | | | | | | | |
| 3.1 | | | | Do you have previous pregnancy history? | | | | | | 1.Yes  2.No | If No to 3.4 | | | | |
| 3.4 | | | | Do you need to have a child in the future? | | | | | | 1. Yes 2. No | If no 3.6 | | | | |
| 3.5 | | | | If yes for 3.4,when do you want to have? | | | | | | 1. in one year  2. after one year  3. 2-3 years  4.3-5 years  5. After five years |  | | | | |
| 3.6 | | | | | Have you ever done abortion Previously | | | | | 1. Yes  2. No | If no 3.9 | | | | |
| 3.7 | | | | | If Yes, how many times with the current one? | | | | |  |  | | | | |
| 3.8 | | | | | What service do you need today? | | | | | 1.safe abortion care service(SAC)  2.post abortion care service(PAC) | If PAC to 3.11 | | | | |
| 3.9 | | | | | If your answer for question No 3.9 is SAC what is the  reason? | | | | | 1.Rape  2.Incest  3.Medical condition  5.Unwanted/Unplanned pregnancy  6. Others. (Specify) |  | | | | |
| 3.10 | | | | | If your answer for question No 3.9 is PAC, how did the abortion started? | | | | | 1.taking drug without prescription  2.Spontanoeus  2.priscribed by traditional healer  3.specify others |  | | | | |
| 4 Decision making related factors | | | | | | | | | | | | | | | |
| 4.1 | | Who is the decision maker in your household to seek care from modern health institution? | | | | | | | 1.Both of us  2.My husband  3.My self | | |  | | | |
| 4.2 | | Does your husband support you to use FP? | | | | | | | 1.Yes  2.No | | |  | | | |
| 5 Knowledge related questions | | | | | | | | | | | | | | | |
| 5.1 | | Do you know modern family planning methods | | | | | | 1. Implants 2. IUCD 3. Injectable 4. Pills 5. Condom 6. Emergency contraception 7. Permanent methods | | |  | | | | |
| 5.2 | | Do you know the importance of post abortion family planning? | | | | | | 1.to prevent recurrent abortion  2. to prevent infection  3.to prevent unwanted pregnancy  4.to prevent heavy bleeding  5.to prevent recurrent abortion related infertility  5. I don’t know  6.others specify | | |  | | | | |
| 5.3 | | Do you know when to use FP after abortion service? | | | | | | 1.Immediately  2.After a Month  3.I don’t know | | |  | | | | |
| 5.4 | | Do you know the recommended duration that a woman should wait before becoming pregnant again after abortion? | | | | | | 1.Immediately  2.weeks  3.after six Months  4. After a year  5.I don’t know | | |  | | | | |
| 5.5 | | Do you know the time after abortion that a woman is at risk of getting pregnant again? | | | | | | 1. within one week  2. between 1 & 2 wks  3. After a month  4. I don’t know | | |  | | | | |

| 6. **Attitude related questions towards service delivery** | | | | | |
| --- | --- | --- | --- | --- | --- |
|  |  | Disagree | Neutral | Agree |  |
| 6.1 | Waiting time at health facility to get the service is short |  |  |  |  |
| 6.2 | The payment to get the Service is fair |  |  |  |  |
| 6.3 | Post abortion family planning provider who work in the center is knowledgeable |  |  |  |  |
| 6.4 | Post abortion family planning provider who work in the center is skillful |  |  |  |  |
| 6.5 | Post abortion family planning provider gives understandable information regarding post abortion family planning |  |  |  |  |
| 6.6 | Post abortion family planning provider was respectful during provision of the service |  |  |  |  |
| 6.7 | Post abortion family planning provider give enough time for discussion |  |  |  |  |
| 6.8 | Post abortion family planning provider permits the mother to decide by her own without oppression |  |  |  |  |
| 6.9 | Confidentiality was assured |  |  |  |  |
| 6.10 | Privacy was assured during the service delivery |  |  |  |  |

**READ: Thank you for taking time to answer these questions.**

**I will accept if you have any suggestion or question.**

**Thank you again for your cooperation!**

**Interview guide questions**

1. What do you think about post abortion family planning in your facility?

Probe: A. what is the relevance of providing post abortion family planning service in your institution?

1. What do you think about the training of PAFP of health professionals in your facility? Probe: A. Do you think that your institution have enough trained personnel as per the client flow?
2. How do you think the ongoing in service training among the health professionals?
3. What do you think about difficulties available in utilizing post abortion family planning in your facility or elsewhere

Probe: A. Do you think that lack of trained personnel is a problem which can hinder post abortion family planning service in your institution?

B. Do you think that your institution have lack of supply/equipment that can hinder the service of post abortion family planning? (Please explain each problem in detail)

C. Do you think service provider related problem can affect the service provision of post abortion family planning in your institution? (Can you explain each problem in detail?)

D. Do you think client related problems can affect the service provision of post abortion family planning in your institution? (Explain each problem in detail)

1. What suggestions you may give to improve post abortion family planning services in this facility or elsewhere
2. What do you think about facility based solutions to improve post abortion family?
3. What do you think about service provider related solutions to improve post abortion family?
4. What is your opinion about client based related to improve the post abortion family service

Thank you for your time and contribution!!!

Contact address Phone no 0934237207

Email tizita23t@gmail.com

**POST ABORTION FAMILY PLANNING COUNSELLING OBSERVATION CHECKLIST**

**Observation ID number: _________ Health Facility Type: ______________ Ownership ___________Code Number __________ Date of Observation: Date__________**

1. Provider providing the Counseling session:

1. Nurse

2. Nurse- Midwife

3. Health officer

4. General practitioner

5. Gyn/Obs specialist

6. Other __________________

**2.** Sex of Provider: **1. Female 2. Male**

**C. For post abortion Family planning**

1. Client status: 1. New 2. Revisit 3. Undetermined

| **2. Did the service provider** | **Yes** | **No** | **Remark** |
| --- | --- | --- | --- |
| 1. Ask open-ended questions? |  |  |  |
| 1. Encourage client to ask questions |  |  |  |
| 1. Treat client with respect |  |  |  |
| 1. See client in private |  |  |  |
| 1. Discuss a return visit |  |  |  |
| 1. Ask client her concerns with any method |  |  |  |
| 1. Use visual aids |  |  |  |
| 1. Use client record |  |  |  |
| 1. Assure client of confidentiality |  |  |  |
| **3. Good Client provider interaction markers (Mark all that done by the service provider satisfactorily)** | | | **“** √ **”** |
| 1. Establish rapport: | | |  |
| 1. Assess the woman’s needs: | | |  |
| 1. Explain human reproduction: | | |  |
| 1. Ask if the woman desires to delay or prevent future pregnancy: | | |  |
| 1. Assess the woman’s individual situation: | | |  |
| 1. Explain characteristics of available methods: | | |  |
| 1. Help the woman choose the method: | | |  |
| 1. Ensure that the woman understands how the method she selected works: | | |  |
| 1. Methods of choice for PAFP and Medical eligibility criteria | | |  |
