# Supplementary material for: Post-abortion family planning utilization and associated factors in health facilities of Wolaita Zone, Southern Ethiopia: Mixed study
Source: PLoS One. 2022 Jun 3;17(6):e0267545. doi: 10.1371/journal.pone.0267545 (PMC9165889; doi:10.1371/journal.pone.0267545)
Supplement: S2 File — (DOCX) [file pone.0267545.s002.docx]

**የፍቃድ ፎርም**

ርዕስ፡ ከፅንስ ማቋረጥ በኋላ የቤተሰብ ምጣኔ አጠቃቀም እና ተያያዥ ምክንያቶች በጤና ተቋማት፡ በወላይታ ዞን: ደቡብ ኢትዮጵያ. ቅይጥ ጥናት

የዋና መርማሪ ስም፡ ትዝታ ተክሌ (ቢኤስሲ)

የድርጅቱ ስም፡- አርባ ምንጭ ዩኒቨርሲቲ

መግቢያ፡-

ሰላምታ፡

ሰላም! ስሜ ___________________በአርባ ምንጭ ዩኒቨርሲቲ የምርምር ቡድን ውስጥ እየሰራሁ ነው ፣ልያናግሩኝ ስለተስማሙ አመሰግናለሁ። ይህ ጥናት በመውለድ ዕድሜ ላይ ያሉ ሴቶች ከውርጃ በኋላ የቤተሰብ ምጣኔ በጤና ተቋማት ውስጥ እንዲጠቀሙ የሚያበረታቱ ወይም ተስፋ የሚያስቆርጡ ሁኔታዎችን ለመለየት የተከናወነ ነው። ጥናቱ በቀጥታ የመራቢያ ዕድሜ ላይ ካሉ ሴቶች ጋር የተያያዘ በመሆኑ በዚህ ጥናት ላይ ለመሳተፍ ከተመረጡት ሴቶች መካከል በመሆንዎ በዚህ ጥናት ላይ እንዲሳተፉና ከናንተ የሚጠበቀውን መረጃ እንድታደርሱ በትህትና እንጠይቃለን።

ከውርጃ በኋላ የቤተሰብ ምጣኔ አጠቃቀም ጋር የተያያዙ አንዳንድ የግል ጥያቄዎችን ልጠይቅዎ ነው። የግድ ትክክል ወይም የተሳሳተ መልስ የለም። በተቻለ መጠን በነፃነት እና በሙሉ እምነት አስተያየትዎን እንዲያካፍሉ እጠይቃለሁ። የምንችለውን ያህል የምላሾችህን ሚስጥራዊነት እንጠብቃለን። ፊርማዎ እንጂ ስምዎ በዚህ ቅጽ ላይ አይጻፍም፤ ከሚነግሩኝ ከማንኛውም መረጃ ጋር በተያያዘም ፈጽሞ ጥቅም ላይ አይውልም።

በዚህ ጥናት ላይ ለመሳተፍ ምንም አይነት ክፍያ አይሰጥዎትም። በዚህ የምርምር ፕሮጀክት ውስጥ መሳተፍ ምንም አይነት አደጋ የለዉም። በዚህ ጥናት ውስጥ ያለዎት ተሳትፎ ሙሉ በሙሉ በፈቃደኝነት ላይ የተመሰረተ ነው እና ከመሳተፍ የመከልከል መብት አለዎት። በቃለ መጠይቁ ላይ ለመሳተፍ ወይም ላለመሳተፍ ያደረጉት ውሳኔ በዚህ ተቋም ውስጥ በሚያገኙት የጤና እንክብካቤ ላይ ተጽእኖ አይኖረውም። መመለስ ለማይፈልጓቸውን ጥያቄዎች መልስ መስጠት አይጠበቅብዎትም፣ እና ይህን ቃለ መጠይቅ በሚፈልጉበት ጊዜ ማቆም ይችላሉ።

ለእነዚህ ጥያቄዎች የሚሰጡት ትክክለኛ መልስ በአገልግሎቶቹ ላይ ስላለው ችግር/ክፍተቶችን ለማሻሻል ይረዳናል። በዚህ ቃለ መጠይቅ ላይ ተሳትፎዎን በጣም እናደንቃለን። ከ10-15 ደቂቃዎች ይወስዳል

ስለ ጥናቱ ማናቸውም ጥያቄዎች ወይም ስጋቶች ወይም ተጨማሪ ማብራሪያ ከፈለጉ በሚከተለው አድራሻ ማግኘት ይችላሉ።

ስም፡ ትዝታ ተክሌ ስልክ ቁጥር፡ 0934237207 ኢሜል፡tizita23t@gmail.com

በዚህ ጥናት ለመሳተፍ ፈቃደኛ ከሆናችሁ፣ ለመሳተፍ ፈቃደኛ ነህ?

አዎ --------------- አይ -----------------

አዎ ከሆነ፣ ፊርማ_________________ ከዚያ ቃለ መጠይቁን ይቀጥሉ

የለም ከሆነ አመስግኑ እና ቃለ መጠይቁን አቁሙ

የቃለ-መጠይቅ ጠያቂው ስም___________-ፊርማ______________የቃለ መጠይቁ ቀን__________________-

የሱፐርቫይዘሩ ስም _______________ፊርማ __________________ ቀን ________________

ለድህረ ዉርጃ የወሊድ መከላከያ አገልግሎት ተደራሽነትና ጥራት ማነቆ የሆኑ ምክንያቶችን ለመለየት ለድህረ ዉርጃ አገልግሎት ተጠቃሚዎች የተዘጋጀ መጠይቅ

| ተ.ቁ | ጥያቄ | | መልስ | | | ማስታወሻ |
| --- | --- | --- | --- | --- | --- | --- |
| 1. የማህበራዊና ኢኮኖሚያዊ ጥያቄዎች | | | | | | |
| 1 | እድሜ | |  | | |  |
| 2 | የመኖሪያ ቦታ | | 1 ከተማ  2 ገጠር | | |  |
| 3 | የጋብቻ ሁኔታ | | 1 ያላገባች  2 ያገባች  3 የተለያየች( በሞት/በፍቺ) | | |  |
| 4 | ሀይማኖት | | 1 ኦርቶዶክስ  2 ፕሮቴስታንት  3 ሙስሊም  4 ካቶሊክ  5 ሌላ | | |  |
| 5 | የወር ገቢ (በብር) | |  | | |  |
| 6 | የትምሀርት ደረጃሽ | | 1 ያልተማረች  2 አንደኛ ደረጃ የተማረች  3 ሁለተኛ ደረጃ የተማረች  4 ሶስተና ደረጃና ከዛ በላይ የተማረች | | |  |
| 7 | የባለቤትሽ/ የጓደኛሽ የትምህርት ደረጃ | | 1 ያልተማረ  2 አንደኛ ደረጃ የተማረ  3 ሁለተኛ ደረጃ የተማረ  4 ሶስተና ደረጃና ከዛ በላይ የተማረ | | |  |
| 8 | የስራ ሁኔታ | | 1 የቤት እመቤት  2 የመንግስት ሰራተኛ  3 መንገስታዊ ያልሆኑ ድርጅቶች ሰራተኛ  4 ተማሪ  5 የቀን ሰራተኛ  6 ነጋዴ  7 ሌላ | | |  |
| 1.9 | የባለቤትሽ/ የጓደኛሽ የስራ ሁኔታ | | 1 የቤት እመቤት/ገበሬ  2 የመንግስት ሰራተኛ  3 መንገስታዊ ያልሆኑ ድርጅቶች ሰራተኛ  4 ተማሪ  5 የቀን ሰራተኛ  6 ነጋዴ  7 ሌላ | | |  |
| 2. የወሊድ መከላከያ አገልግሎትን በተመለከተ | | | | | | |
| 2.1 | ከዚህ ቀደም ስለ ወሊድ መከላከያ የምክር አገልግሎት ወስደው ያውቃሉ? | | 1 አዎ  2 አይ | | |  |
| 2.2 | ከዚህ ቀደም የወሊድ መከላከያ ተጠቅመው ያዉቃሉ? | | 1 አዎ  2 አይ | | |  |
| 2.3 | ዛሬ ከጤና ባለሙያው ጋር በወሊድ መከላከያ ዙሪያ ምክክር አድርጋችኋል? | | 1 አዎ  2 አይ | | |  |
| 2.4 | ዛሬ የወሊድ መከላከያ ወስደዋል? | | 1 አዎ  2 አይ | | | መልሱ አይ ከሆነ ወደ ተራ ቁጥር 2.7 |
| 2.5 | የትኛዉን አይነት የወሊድ መከላከያ ነው የወሰዱት? | | 1 የሚዋጥ ኪኒን  2 በመርፌ የሚሰጥ  3 በክንድ የሚቀበር  4 ማህፀን ውስጥ የሚቀመጥ  5 ኮንዶም  6 ማህፀን ማስቋጠር | | |  |
| 2.6 | የወሰዱት የወሊድ መከላለያ የሚፈልጉትን መርጠው ነው? | | 1 አዎ  2 አይ | | |  |
| 2.7 | የወሊድ መከላከያ ካልወሰዱ ምክንያትዎ ምንድነው? | | 1 መረጃውን ስላላገኘው  2 በቅርቡ ማርገዝ ስለምፈልግ  3 የምፈልገውን የወሊድ መከላከያ ስላላገኘው  4 ባለቤቴ ሩቅ ሀገር ስላለ  5 ከእንግዲህ ከግብረስጋ ግኑኝነት መታቀብ ስለምፈልግ  6 የጎንዮሽ ጉዳቱን ፈርቼ  7 ሌላ | | |  |
| 3. ስነ ተዋልዶ ጤና ጋር በተያያዘ | | | | | | |
| 3.1 | ከዚህ በፊት አርግዘው ያውቃሉ? | | 1 አዎ  2 አይ | | |  |
| 3.2 | ወደፊት ተጨማሪ ልጅ እንዲኖርዎ ይፈልጋሉ? | | 1 አዎ  2 አይ | | |  |
| 3.3 | ከፈለጉ መቼ እንዲኖርዎ ነው የሚፈልጉት? | | 1 በአንድ አመት ውስጥ  2 ከአንድ አመት በላይ | | |  |
| 3.4 | ከዚህ በፊት ውርጃ አጋጥምዎት ያውቃል? | | 1 አዎ  2 አይ | | | አይ ከሆነ ውድ ተ ቁ 3.6 |
| 3.5 | ከሆነ ከዚህ ጋር ስንት ጊዜ ነው ያጋጠምዎት? | |  | | |  |
| 3.6 | ዛሬ የትኛዉን አይነት የውርጃ አገልግሎት ለመጠቀም ነው የመጡት? | | 1 የውርጃ አገልግሎት  2 የድህረ ውርጃ አገልግሎት | | | የድህረ ውርጃ አገልግሎት ከሆነ ወደ ተ.ቁ 3.8 |
| 3.7 | መልስዎ ለጤናማ የውርጃ አገልግሎት ከሆነ ፅንሱን ለማጨናገፍ የፈለጉበት ምክንያት ምንድነው? | | 1 ተደፍሬ ስለፀነስኩ  2 ከዘመድ ስለፀነስኩ  3 ለህይወቴ አስጊ የጤና እክል ስለገጠመኝ  4 ያልተፈለገ እርግዝና ስለገጠመኝ  5 ሌላ | | |  |
| 3.8 | መልስዎ የድህረ ውርጃ አገልግሎት ከሆነ ውርጃው እንዴት ነው የጀመርዎት? | | 1 የባህላዊ መድሃኒት ወስጄ  2 ራሴ ዝም ብዬ ብዙ መድሃኒት ወስጄ  3 በራሱ ነው የጀመረኝ  4 ሌላ | | |  |
| 4. ዉሳኔ ሰጪነትን በተመለከተ | | | | | | |
| 4.1 | በቤትዎ/ በግኑኝነትዎ የጤና አገልግሎት ለመጠቀም ዉሳኔ የሚሰጠው ማነው? | | 1 እኔና ባለቤቴ/ጓደኛዬ ተመካክረን  2 ባለቤቴ ብቻውን  3 እኔ ብቻዬን | | |  |
| 4.2 | ባለቤትሽ የወሊድ መከላከያ እንድትወስጂ ይደግፍሻል/ይገፋፋሻል | | 1 አዎ  2 አይ | | |  |
| 5. ከድህረ ውርጃ ወሊድ መከላከያ እውቀትን በተመለከተ | | | | | | |
| 5.1 | ዘመናዊ የወሊድ መከላከያ አይነቶችን ያውቃሉ? | | 1 ክንድ ስር የሚቀበር  2 በመርፌ የሚሰጥ  3 ማህፀን ውስጥ የሚቀመጥ  4 የሚዋጥ ኪኒን  5 ኮንዶም  6 ዘላቂ መከላከያ(ማህፀን ማስቋጠር)  7 የድንጋተኛ እርግዝና መከላከያ | | |  |
| 5.2 | ድህረ ውርጃ መከላከያ ጥቅሞችን ያዉቃሉ? | | 1 ተደጋጋሚ ውርጃን ለመከላከል  2 በውርጃ ምክንያት የሚከሰት ማመርቀዝን ለመከላከል  3 ያልተፈለገ እርግዝናን ለመከላከል  4 በውርጃ ምክንያት የሚከሰት ከፍተኛ የሆነ የድም መፍሰስን ለመከላከል  5 በትደጋጋሚ ውርጃ ምክንያት የሚከሰት መካንነትን ለመከላከል  6 ሌላ | | |  |
| 5.3 | ከውርጃ አግልግሎት በኋላ መቼ ነው የወሊድ መከላከያ መጠቀም የሚገባው? | | 1 ወዲያው ከአገልግሎቱ በኋላ  2 ከወር በኋላ አገግመን ስንመለስ  3 ሌላ | | |  |
| 5.4 | አንዲት እናት ከውርጃ በኋላ በምን ያህል ጊዜ ውስጥ ነው ልታረግዝ የምትችለው | | 1 ከሁለት ሳምንት በኋላ  2 ከወር በኋላ  3 ሌላ | | |  |
| 5.5 | አንዲት እናት ከውርጃ በኋላ ከምን ያህል ጊዜ በኋላ ነው ድጋሚ እንድታረግዝ የሚመከረው? | | 1 ወዲያዉኑ  2 ከአንድ ወር ብኋላ  3 ከስድስት ወር በኋላ  4 ሌላ | | |  |
| 6. የድህረ ዉርጃ ወሊድ መከላከያ አገልግሎት አመለካከትን በተመለከተ | | | | | | |
|  |  | አልስማማም | | ሀሳብ የለኝም | እስማማለሁ |  |
| 6.1 | አገልግሎቱን ለመጠቀም የጠበኩት ጊዜ አጭር ነው |  | |  |  |  |
| 6.2 | አገልግሎቱን ለመጠቀም የከፈልኩት ክፍያ ትንሽ ነው |  | |  |  |  |
| 6.3 | አገልግሎቱን በሰጠኝ ባላሙያ ዕውቀት እተማመናለሁ |  | |  |  |  |
| 6.4 | አገልግሎቱን በሰጠኝ ባላሙያ ብቃት እተማመናለሁ |  | |  |  |  |
| 6.5 | አገልግሎቱን የሰጠኝ ባላሙያ በሚገባኝ ቋንቋ መረጃውን ሰቶኛል |  | |  |  |  |
| 6.6 | አገልግሎቱን የሰጠኝ ባላሙያ በአክብሮት አስተናግዶኛል |  | |  |  |  |
| 6.7 | አገልግሎቱን የሰጠኝ ባላሙያ ለመወያየት በቂ ጊዜ ሰቶኛል |  | |  |  |  |
| 6.8 | አገልግሎቱን የሰጠኝ ባላሙያ ሳይጫነኝ ምርጫዬን አክብሮልኛል |  | |  |  |  |
| 6.9 | የመረጃዎቼ ምስጢራዊነት ይጠበቅልኛል |  | |  |  |  |
| 6.10 | በአገልግሎት አሰጣት ውስጥ ግላዊነቴ ተጠብቆልኛል |  | |  |  |  |

ይነበብ፡

ጊዜዎትን ስለሰጡን እጅግ በጣም እናመሰግናለን። ማንኛውም አይነት ጥያቄ ወይም አስተያየት ካሎት ለመቀበል ዝግጁ ነኝ።

ከሌሎት በድጋሚ አመሰግናለሁ።

አድራሻ፡ ስ.ቁጥር:- 0934237207

ኢሜይል:- tizita23t@gmail.com

ለጤና ባለሙያዎች የተዘጋጀ መጠይቅ

1. በጤና ተቋምዎ ስለሚሰጠው የድህረ ዉርጃ የወሊድ መከላከያ አገልግሎት ምን አስተያየት አሎት?

A. በተቋምዎ ውስጥ ከፅንስ ማስወረድ በኋላ የቤተሰብ ምጣኔ አገልግሎት መስጠት ያለው ጠቀሜታ ምን ይመስልዎታል

1. በጤና ተቋምዎ ውስጥ ከድህረ ዉርጃ ወሊድ መከላከያ ጋር የተያያዘ ለባለሞያውች የሚሰጥ የክህሎት ማዳበር ስልጠናዎች ተደራሽነት ምን ይመስላል?
2. የሰለጠነ ባለሙያ ቁጥር ጋር ከተገልጋዩ ጋር የሚመጣጠን ብለው ያምናሉ?
3. በጤና ተቋምዎ በስራ ላይ ስልጠናዎች እንዴት ይገልፁታል?
4. በጤና ተቋምዎ ውስጥ ለድህረ ውርጃ አገልግሎት ጥራት ማነቆ የሆኑ ነገሮች ምንድናቸው?(የሰለጠነ ባለሙያ፣ የቁሳቁስ እጥረት፣ የገንዘብ እጥረት፣ ከባለሙያ ጋር የተያያዘ ችግር፣ ከተጠቃሚ ጋር የተያያዘ ችግር)
5. በጤና ተቋምዎ ውስጥ የሰለጠነ የሰው ሃይል እጥረት የድህረ ዉርጃ የወሊድ መከላከያ አገልግሎት ማነቆ ነው ብለው ያስባሉ?
6. የእርስዎ ተቋም ከውርጃ በኋላ የቤተሰብ ምጣኔ አገልግሎትን የሚያደናቅፍ የአቅርቦት/የመሳሪያ እጥረት አለበት ብለው ያስባሉ? (እባክዎ እያንዳንዱን ችግር በዝርዝር ያብራሩ)
7. ከአገልግሎት ሰጪው ጋር የተያያዘ ችግር በተቋምዎ ውስጥ ከውርጃ በኋላ የቤተሰብ ምጣኔ አገልግሎት አሰጣጥ ላይ ተጽእኖ ሊያሳድር ይችላል ብለው ያስባሉ? (እባክዎትን እያንዳንዱን ችግር በዝርዝር ያብራሩልን?)
8. ከደንበኛ ጋር የተያያዙ ችግሮች በተቋምዎ ውስጥ ከውርጃ በኋላ የቤተሰብ ምጣኔ አገልግሎት አቅርቦት ላይ ተጽእኖ ሊያሳድሩ ይችላሉ ብለው ያስባሉ? (እያንዳንዱን ችግር በዝርዝር አስረዳ)
9. በተቋም ዉስጥ ከውርጃ በኋላ የቤተሰብ ምጣኔ አገልግሎቶችን ለማሻሻል ምን አይነት ጥቆማዎችን መስጠት ይችላሉ?
10. ከውርጃ በኋላ የቤተሰብ ምጣኔ አገልግሎትን ለማሻሻል በተቋሙ ላይ ስለተመሰረቱ መፍትሄዎች ምን ያስባሉ?
11. ከውርጃ በኋላ የቤተሰብ ምጣኔ አገልግሎትን ለማሻሻል ከጤና ባለሙያዎች ጋር ተያያዥ መፍትሄዎችን ምን ያስባሉ?
12. ከውርጃ በኋላ የቤተሰብ ምጣኔ አገልግሎትን ለማሻሻል ከደንበኛ ጋር ተያያዥ መፍትሄዎችን ምን ያስባሉ?

**ጊዜዎትን ሰተው ስላሳዩን ቀና ትብብር እጅግ በጣም እናመሰግናለን። ማንኛውም አይነት ጥያቄ ወይም አስተያየት ካሎት ለመቀበል ዝግጁ ነኝ። ከሌሎት በድጋሚ አመሰግናለሁ።**

**አድራሻ፡** ስ.ቁጥር:- 0934237207

ኢሜይል:- tizita23t@gmail.com
